# In-silico study of asymmetric remodeling of tumors in response to external biochemical stimuli

**DOI:** 10.1038/s41598-022-26891-8

**Published:** 2023-01-18

**Authors:** Meitham Amereh, Mohsen Akbari, Ben Nadler

**Affiliations:** 1 Department of Mechanical Engineering, University of Victoria, Victoria, BC Canada; 2 Laboratory for Innovations in MicroEngineering (LiME), Department of Mechanical Engineering, University of Victoria, Victoria, BC Canada; 3 Centre for Advanced Materials and Related Technologies (CAMTEC), University of Victoria, Victoria, BC Canada; 4 Biotechnology Center, Silesian University of Technology, Akademicka, Gliwice, Poland; 5 Terasaki Institute for Biomedical Innovations, Los Angeles, CA USA

**Keywords:** Cancer, Mathematics and computing

## Abstract

Among different hallmarks of cancer, understanding biomechanics of tumor growth and remodeling benefits the most from the theoretical framework of continuum mechanics. Tumor remodeling initiates when cancer cells seek new homeostasis in response to the microenvironmental stimuli. Cells within a growing tumor are capable to remodel their inter- and intra-connections and become more mobile to achieve a new homeostasis. This mobility enables the tumor to undergo large deformation. In this work, we studied the remodeling of homogeneous tumors, at their early stage of growth, in the context of continuum mechanics. We developed an evolution law for the remodeling-associated deformation which correlates the remodeling to a characteristic tensor of external stimuli. The asymmetric remodeling and the induced mechanical stresses were analyzed for different types of biochemical distributions. To experimentally investigate the model, we studied the remodeling of human glioblastoma (hGB) tumoroids in response to the gradient of nutrients. Using a tumoroid-on-a-chip platform, the degree of remodeling was estimated for the ellipsoidal tumoroids over time. It was observed that higher gradient of nutrients induces higher degree of ellipticity suggesting that the gradient of nutrient is a characteristic property of nutrient distribution that derives the remodeling. We also showed that remodeling gives rise to heterogeneity in cell distribution forming circumferentially aligned cells within the tumors. Compared to the existing studies on tumor growth, our work provides a biomechanical module that relates the remodeling to biochemical stimuli, and allows for large deformation. It also includes experimental component, a necessary but challenging step, that connects the theory and reality to evaluate the practicability of the model.

## Introduction

Cancer has been recognized as one the most challenging problems in biology and medicine. Aggressive tumors are lethal type of cancers characterized by high genomic instability, rapid progression, invasiveness and therapeutic resistance^1^. Their behavior involves complicated molecular biology and consequential dynamics. Diversity of mechanisms involved in cancer progression makes the treatment strategies inevitable to approach from different areas of knowledge. Although tremendous effort has been devoted to developing new therapeutics, there is still a huge need for new insights into the less known aspects of tumors such as evolution of heterogeneity, biomechanical responses, etc.^2^. Mathematical modeling, and continuum mechanics in particular, can play a pivotal role in better understanding the complex behavior of tumors by providing quantitative predictions of biological processes involved in tumor growth and invasion and helping to interpret complicated mechanical interactions between tumor and microenvironment^3^. Having insight into mechanical responses of a growing tumor can contribute in development of cancer therapeutic approaches. Mathematical oncology, for instance, with a broad scope including theoretical studies and mathematical models used to design clinical trials, has become a dominant field of research in personalized medicine^4^. In-silico cancer models have a great potential to help improving therapeutic efficacy and drug design and delivery in clinical oncology. Applications of continuum based in-silico models in simulating the growth of vascularised solid tumours have been reviewed in^5^.

The pathophysiology of aggressive tumors is strongly affected by the extracellular cues such as nutrients, growth factors, oxygen and stresses^6^. For instance, a growing tumor can change its homeostasis in response to nutrient supply via different mechanisms^7^. One of such mechanisms is remodeling by breaking and making adherent junctions^8^. During this process, the tumor stops the rapid proliferation and begins to remodel its shape to preserve the homeostatic equilibrium state. To reach this, the tumor in turn upregulates epithelial to mesenchymal transit-inducing transcription factors (EMT-TFs)^9^. These EMT-TFs are involved in various signaling cascades which are often associated with tumor invasiveness and malignancy^10^.

The growth of solid tumor spheroids has been mathematically studies from different perspectives such as continuum, discrete and hybrid (continuum-discrete) methods^11–14^. These models built upon partial differential equations (PDEs) for mass conservation and evolution of the tumor boundary, considering the concentrations of cells and nutrients as the main variables that define the local proliferation/death of cells. In addition, the stability of the radially symmetric growth to asymmetric perturbation is explored using the same mathematical framework^15,16^. These analyses indicated that the spherical configuration of tumor spheroids are naturally unstable to asymmetric perturbations. Despite the success in exposition of the role of properties such as surface tension and rate of proliferation in the stability of growth, they lack description of several components including the the mechanical stress distribution, anisotropic growth, the role of mechanical forces on the net tumor growth and mechanical interaction between tumor and extracellular matrix (ECM). These aspects of tumor progression are exclusively investigated in the framework of continuum mechanics^17,18^. For instance, the influence of anisotropic growth on the overall growth and the resultant stress in avascular tumor spheroids is modeled by introducing a growth tensor^19–21^. Analysis of the radial and circumferential stresses on the degree of anisotropy revealed that growth rate is inversely proportional to the magnitudes of stresses. Anisotropic growth was also studied using a biomechanical model for the growth of spinal tumors^22^. The isotropic vs. anisotropic effects on the evolution of stress and interstitial pressure in intramedullary spinal tumours were predicted which revealed a significant difference in tumors shape. Results of these studies provide a direction to the prediction of different growth patterns, yet missing the remodeling module that allows for cell rearrangement and large deformation. Including experimental component is another necessary but challenging step that connects the theory and reality and evaluates the practicability of the models.

Despite the significance of these studies and the important results they provided, they do not account for the remodeling. Tumor progression is a multi-aspect complex process that includes growth, remodeling, etc. Remodeling is one of the important phenomena that occurs in solid tumors in response to external effects. The remodeling process takes place via different biomechanical rearrangements including cell-cell and cell-ECM adhesion bindings and can give rise to large deformation. Hence, study of the remodeling can help elucidate the dynamics of cell-cell and cell-matrix interactions stimulated by external influences. In this study, we proposed a mathematical framework based on the principles of continuum mechanics to better understand the role of biochemical stimuli in tumor remodeling. A volume-preserving evolution of remodeling was obtained that relates the characteristic tensor of external stimuli to the elastic response of tumor and the associated remodeling deformation. The tumor was taken to be hyperplastic material with Ciarlet strain energy function. The asymmetric remodelling and the induced stress distribution were analyzed for linear and quadratic distribution fields of biochemical stimuli. To experimentally validate the model, the shape of human glioblastoma (hGB) tumoroids exposed to the gradient of nutrient was studied over 6 days, using an open-well tumor-on-a-chip platform. We introduced a gradient of nutrients within a composite hydrogel mimicking the extracellular matrix of hGB, and investigated the asymmetric remodeling of tumoroids in response to the nutrient gradient.

## Biomechanical model

In the theory of material evolution, configurational forces are thermodynamically dual to the evolution rate, driving the growth, remodeling and material evolution^23^. The significance of these forces is explored in different areas of continuum mechanics, including defects^24^, interfaces^25^ and biological materials^26^. From the study of cell morphology, it is observed that a cluster of cells has the ability to break and form new bonds in the microstructure^27–29^. This mechanism is important when tumors are subjected to external stimuli such as mechanical stress and biological signals. From microstructure and continuum mechanics points of view, one may think of such microstructural rearrangement, analogous to plastic deformation, where the energy spent on remodeling of tumor microstructure dissipates, while the tumor conserves the energy associated with small elastic deformations.

### Theory of material evolution

**Figure 1:**
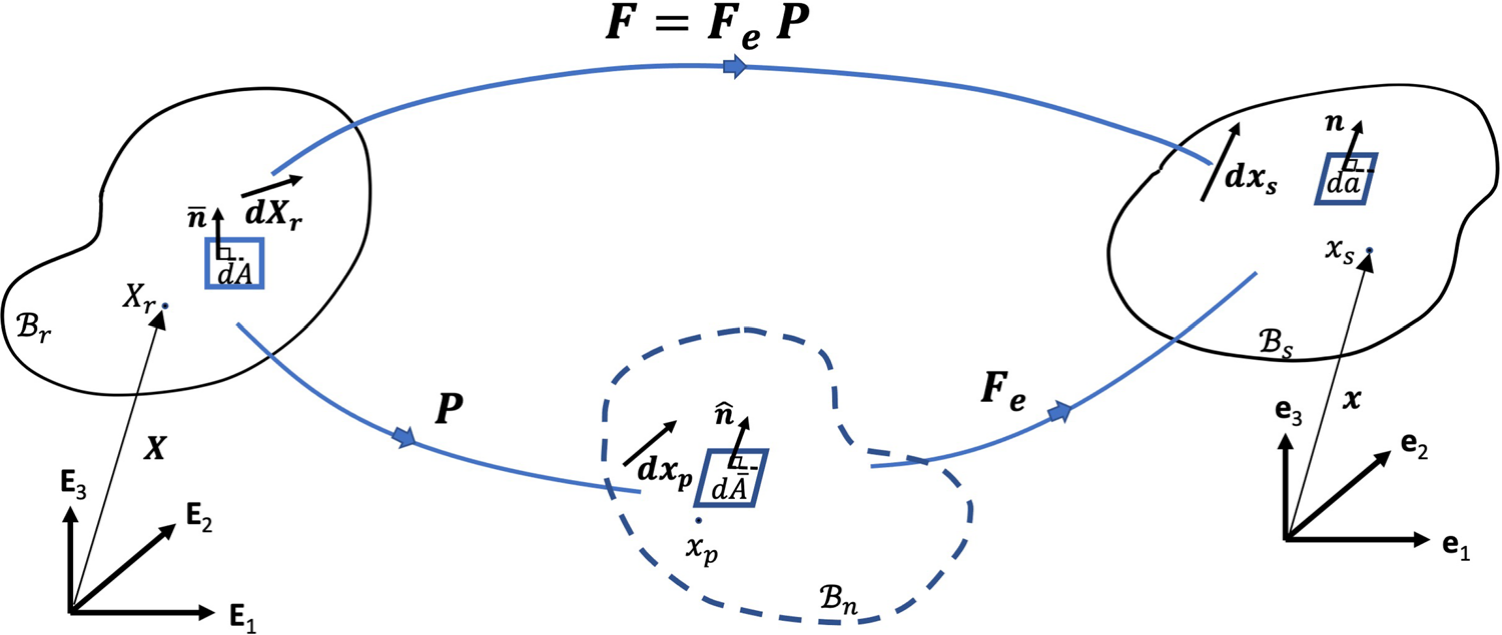
Multiplicative decomposition of the deformation ${\textbf{F}}={\textbf{F}}_{e}\,{\textbf{P}}$. Initial configuration ${\mathscr {B}}_{r}$, natural configuration ${\mathscr {B}}_{n}$ and the current configuration ${\mathscr {B}}_{s}$ together with their area elements and normal vectors are depicted in the figure. ${\textbf{E}}_i$ and ${\textbf{e}}_i$, $i=\{1,2,3\}$, are orthonormal bases in ${\mathscr {B}}_{r}$ and ${\mathscr {B}}_{s}$, respectively.

In this work we adopt the theory of material evolution to capture the time-dependent remodeling responses of tumors^30,31^. We consider the tumor body as a simple evolving material, i.e. defined by their strain energy function, and neglect the thermodynamics effects, such as temperature. The invertible linear deformation gradient ${\textbf{F}}$ is defined to map referential vector ${\textbf{X}}_r$ from the initial configuration ${\mathscr {B}}_{r}$ to the spatial vector ${\textbf{x}}_s$ in the current configuration ${\mathscr {B}}_{s}$, shown in Fig. 1. The multiplicative decomposition of ${\textbf{F}}$ reads ${\textbf{F}}={\textbf{F}}_{e}\,{\textbf{P}}$, where ${\textbf{F}}_{e}$ and ${\textbf{P}}$ are elastic and remodelling deformation tensors, respectively. The remodelling, denoted by ${\textbf{P}}$, is the time-dependent induced invertible linear map from the initial configuration ${\mathscr {B}}_{r}$ to the natural configuration ${\mathscr {B}}_{n}$. The natural configuration is taken to be stress-free and used as a reference configuration for the constitutive law.

We assume that the energy supplied to the system includes mechanical power and other form of power flux, that stimulates remodeling by microstructural rearrangement, and has the dissipation inequality form

1 $$\begin{aligned} \dot{\overline{J\rho \psi }} - J{\textbf{T}}\cdot {\textbf{L}}- J_p \rho {\textbf{S}}\cdot {\textbf{L}}_p \leqslant 0\ \,, \end{aligned}$$

where $\psi$ is free energy per unit mass, $\rho$ is the mass density, $J=\det {\textbf{F}}$, ${\textbf{T}}$ is Cauchy stress tensor, ${\textbf{L}}={\dot{{\textbf{F}}}} {\textbf{F}}^{-1}$ is the velocity gradient, $J_p = \det ({\textbf{P}})$, ${\textbf{S}}$ is the configurations stress per unit mass associated with external stimuli deriving the remodeling. Also, ${\textbf{L}}_p={\dot{{\textbf{P}}}}\,{\textbf{P}}^{-1}$ is the rate of remodeling such that ${\textbf{S}}\cdot {\textbf{L}}_p$ is the remodeling power where the inner product of two second-order tensors is defined as ${\textbf{S}}\cdot {\textbf{L}}_p={\text {tr}}{({\textbf{S}}^T {\textbf{L}}_p)}$. Here, we focus on remodeling while neglecting the growth, that is $J_p =1$ and $\dot{\overline{J\rho }}=0$, hence $\dot{\overline{J\rho \psi }}=J\rho {\dot{\psi }}$.

For elastic material the constitutive law states that strain energy is a function of the deformation $\psi = {\hat{\psi }}({\textbf{F}}_{e})$, which yields ${\dot{\psi }} = {(\partial \psi / \partial {\textbf{F}}_{e}}) \cdot {\dot{{\textbf{F}}}}_{e}$. Now, equation (1) takes the form

2 $$\begin{aligned} J\rho \, { \frac{\partial \psi }{\partial {\textbf{F}}_{e}}} \cdot {\dot{{\textbf{F}}}}_{e} \leqslant J{\textbf{T}}\cdot {\textbf{L}}+ \rho \, {\textbf{S}}\cdot {\textbf{L}}_p\,, \end{aligned}$$

where the partial derivative $\partial A / \partial {\textbf{B}}$ stands for the second-order-tensor-valued spatial derivative of the scalar-valued function *A* with respect to the second-order tensor variable ${\textbf{B}}$. Rearranging (2) into the convenient form yields

$$\begin{aligned} J\rho \, { \frac{\partial \psi }{\partial {\textbf{F}}_{e}}} \cdot {\dot{{\textbf{F}}}}_{e}\leqslant & {} J{\textbf{T}}\cdot ({\dot{{\textbf{F}}}}{\textbf{F}}^{-1}) + \rho \, {\textbf{S}}\cdot {\dot{{\textbf{P}}}}\,{\textbf{P}}^{-1}\\\leqslant & {} J{\textbf{T}}\cdot ({\dot{{\textbf{F}}}}_{e} {\textbf{P}}+ {\textbf{F}}_{e} {\dot{{\textbf{P}}}})({\textbf{P}}^{-1}{\textbf{F}}^{-1}_{e}) + \rho \, {\textbf{S}}\cdot {\dot{{\textbf{P}}}}\,{\textbf{P}}^{-1}\\\leqslant & {} J{\textbf{T}}\cdot ({\dot{{\textbf{F}}}}_{e} {\textbf{P}}{\textbf{P}}^{-1}{\textbf{F}}^{-1}_{e} + {\textbf{F}}_{e} {\dot{{\textbf{P}}}} {\textbf{P}}^{-1} {\textbf{F}}^{-1}_{e} + \rho \, {\textbf{S}}\cdot {\dot{{\textbf{P}}}}\,{\textbf{P}}^{-1}\\\leqslant & {} J{\textbf{T}}{\textbf{F}}^{-T}_{e} \cdot {\dot{{\textbf{F}}}}_{e} + J{\textbf{F}}^{T}_{e}{\textbf{T}}{\textbf{F}}^{-T}_{e} \cdot {\dot{{\textbf{P}}}}\,{\textbf{P}}^{-1} + \rho \, {\textbf{S}}\cdot {\dot{{\textbf{P}}}}\,{\textbf{P}}^{-1}\,,\\ \end{aligned}$$

hence

3 $$\begin{aligned} \left( J{\textbf{T}}{\textbf{F}}^{-T}_{e} - J\rho \, { \frac{\partial \psi }{\partial {\textbf{F}}_{e}}} \right) \cdot \,{\dot{{\textbf{F}}}}_{e} + \left( {\textbf{M}}+ \rho {\textbf{S}}\right) \cdot \,{\textbf{L}}_p\, \geqslant 0\, , \end{aligned}$$

where the Mandel stress is

4 $$\begin{aligned} {\textbf{M}}=J{\textbf{F}}^{T}_{e}{\textbf{T}}{\textbf{F}}^{-T}_{e}\,. \end{aligned}$$

Since ${\dot{{\textbf{F}}}}$ and ${\textbf{L}}_p$ are independent and the elastic part is non-dissipative, inequality (3) requires that

5 $$\begin{aligned}{} & {} \left( J{\textbf{T}}{\textbf{F}}^{-T}_{e} - J\rho \, { \frac{\partial \psi }{\partial {\textbf{F}}_{e}}} \right) \cdot \,{\dot{{\textbf{F}}}}_{e} = 0\, , \, \end{aligned}$$

6 $$\begin{aligned}{} & {} \left( {\textbf{M}}+ \rho {\textbf{S}}\right) \cdot \,{\textbf{L}}_p\, \geqslant 0\, . \end{aligned}$$

To satisfy (5), it is necessary that

7 $$\begin{aligned} {\textbf{T}}= \rho \, { \frac{\partial \psi }{\partial {\textbf{F}}_{e}}} {\textbf{F}}^{T}_{e} \, . \end{aligned}$$

As ${\textbf{L}}_p$ involves pure remodeling, it implies that $\dot{J_p} = {\text {tr}}{{\textbf{L}}_p} = 0$. By (6), it follows that only the divatoric part of $\left( {\textbf{M}}+ \rho {\textbf{S}}\right)$ yields a non-vanishing power, hence, it can be expressed as

8 $$\begin{aligned} \left( {\textbf{M}}+ \rho {\textbf{S}}\right) ^\text {div} \cdot \,{\textbf{L}}_p\, \geqslant 0\, , \end{aligned}$$

where the superscript $\text {div}$ is the deviatoric part of the argument. It follows that the form

9 $$\begin{aligned} {\textbf{L}}_p = \left( {\mathbb {K}} \left( {\textbf{M}}+ \rho {\textbf{S}}\right) \right) ^\text {div}\, , \end{aligned}$$

automatically satisfies (8) given that ${\mathbb {K}}$ is a positive semi-definite fourth order tensor. Finally, the evolution of the remodeling has the form

10 $$\begin{aligned} {\dot{{\textbf{P}}}}= \left( {\mathbb {K}} ({\textbf{M}}+ \rho {\textbf{S}})\right) ^\text {div}\, {\textbf{P}}\, , \end{aligned}$$

where $({\textbf{M}}+ \rho {\textbf{S}})$ is the remodeling stimuli and ${\mathbb {K}}$ represents the anisotropy relationship between the direction of the stimuli and the remodeling.

### Governing equations

For solids undergoing large deformation it is convenient to use the known referential configuration, ${\mathscr {B}}_r$, to express the governing equations, rather than the unknown spatial configuration ${\mathscr {B}}_s$. The referential forms of the conservation of mass and balance of linear momentum are

11 $$\begin{aligned} \rho= & {} J^{-1} \rho _0\,, \end{aligned}$$

12 $$\begin{aligned} \rho _0 {\dot{V}}_R= & {} \rho _0 {\textbf{b}}+ {\text {Div}}(\pmb {\Pi })\,, \end{aligned}$$

where $\rho _0$ and $\rho$ are the spatial and referential densities, respectively, ${\dot{V}}_R$ is the material time derivative of the velocity, ${\textbf{b}}$ is the body force, $\pmb {\Pi }$ is the Piola stress, and ${\text {Div}}$ is referential divergence operator. Next step is to utilize a constitutive strain energy for the tumor mass to relate the deformation to the stress.

Observations suggest that small avascular solid tumors, made of single cell type population, behave as isotropic compressible materials^32,33^. In addition, biological tissues show rate-dependent responses, and hence viscoelastic constitutive equations are used to model their behaviour. However, the solid tumor spheroids have a small relaxation time compared to the time scale of biological evolution such as growth, remodelling, invasion, etc.^34^. Therefore, we consider solid tumor spheroids as hyperelastic isotropic compressible material with strain energy function that depends only on the deformation gradient. It has been shown that the evolution of stress in tumors has limited dependency on the specific hyperelastic constitutive relations^35^. Therefore, we adopt the commonly used Ciarlet strain energy^33^

13 $$\begin{aligned} \psi =\displaystyle \frac{\lambda }{4}\,\bigg [ I_3({\textbf{B}}_{e})-\ln {I_3({\textbf{B}}_{e})}-1\bigg ] + \frac{\mu }{2} \bigg [I_1({\textbf{B}}_{e})-\ln {I_3({\textbf{B}}_{e})}-3\bigg ]\,, \end{aligned}$$

where ${\textbf{B}}_{e}={\textbf{F}}_{e}\,{\textbf{F}}_{e}^T$ is the left Cauchy-Green elastic deformation tensor, $I_1({\textbf{B}}_{e})$, $I_3({\textbf{B}}_{e})$ are the principal invariants of ${\textbf{B}}_{e}$, and $\lambda$ and $\mu$ are Lame’s constants associated with the tumor. Substitution of (13) in (7) gives the Cauchy stress tensor

14 $$\begin{aligned} {\textbf{T}}= \rho \bigg [\mu {\textbf{B}}_{e} + \frac{\lambda }{2}\left( I_3({\textbf{B}}_{e})-1 \right) {\textbf{I}}-\mu {\textbf{I}}\bigg ]\,. \end{aligned}$$

The transformation of the Cauchy stress tensor to the Piola stress tensor is

15 $$\begin{aligned} \pmb {\Pi } = J\, {\textbf{T}}\, {\textbf{F}}^{-T}\,. \end{aligned}$$

Finally, the spatial boundary condition is

16 $$\begin{aligned} {\textbf{T}}\,{\textbf{n}}= {\textbf{t}}, \end{aligned}$$

where ${\textbf{n}}$ is the normal and ${\textbf{t}}$ is the boundary traction. The boundary normal can be calculated using Nanson’s equation expressed as

17 $$\begin{aligned} {\textbf{n}}= \left( {\bar{{\textbf{n}}}} \cdot {\textbf{C}}^{-1}\, {\bar{{\textbf{n}}}} \right) ^{-\frac{1}{2}} {\textbf{F}}^{-T} {\bar{{\textbf{n}}}}\,. \end{aligned}$$

where ${\bar{{\textbf{n}}}}$ is the boundary normal in the reference configuration and ${\textbf{C}}$ is right Cauchy-Green deformation tensor. Equations (12) and (16) together with the evolution of remodeling (10) form the governing equations. In the next section, we discuss the solution procedure.

### Theoretical study of tumor remodelling

The evolution of remodeling and the governing equation for the stress were presented in the previous section. In this section, we extend the correlation between the remodeling and the nutrient distribution. To that end, it is necessary to associate configuration stress ${\textbf{S}}$ with the effect of external stimuli.

Gradient of biochemicals such as nutrient and chemo- attractants are among the important biological stimuli that can evoke tumor response. For instance, in chemotaxis, cells change their morphology, break E-cadherin junctions and move toward the gradient of chemo-attractant^36^. Another example is the ability of tumors to remodel their shape in response to external forces that relieves the stress and preserves the homeostatic equilibrium state^8^. A tumor, that is deprived from sufficient nutrients can expand its boundaries by remodeling the microstructure (i.e. binding and unbinding the inter- and intra-cellular junctions) in favor of reaching new source. Here, we propose that the gradient of nutrient is the main property of nutrient distribution that governs the remodeling. Hence, we take the characteristic tensor associated to the biochemical stimuli in the spatial configuration to be aligned with gradient of nutrients in the form

18 $$\begin{aligned} {\textbf{s}}= {\text {grad}}c \otimes {\text {grad}}c \,, \end{aligned}$$

where *c* is the nutrients concentration. The pullback of this stimuli into the natural configuration is

19 $$\begin{aligned} {\textbf{S}}= J{\textbf{F}}_{e}^{T}\,{\textbf{s}}\,{\textbf{F}}_{e}^{-T} \,. \end{aligned}$$

Here we take the evolution of the remodeling to be isotropic, that is, the remodeling is aliened with the stimuli $\left( {\textbf{M}}+\rho {\textbf{S}}\right)$

20 $$\begin{aligned} {\mathbb {K}}= a {\mathbb {I}} \, , \end{aligned}$$

where $a\ge 0$ determines the intensity of stimuli that drives the evolution of the remodeling. Also, this equation shows that the remodeling tensor is aligned with the tensor of stimuli, and ${\mathbb {I}}$ is the forth order identity. We further assume that there is a constant supply of nutrients and the time scale of the nutrient diffusion is much faster then the evolution of the tumor. Therefore, we take the nutrients field to be time-independent.

Here, we use the mathematical model presented in “Biomechanical model” to study the remodeling of tumor spheroids in response to gradient of external stimuli that is generated using tumor-on-a-chip platform. For simplicity, we consider two-dimensional geometry subjected to gradient of nutrients. The tumor is initially circular, hence ${\textbf{X}}=R{\hat{{\textbf{E}}}}_R\,(\Theta )$, where $\{R, \Theta \}$ and $\{{\textbf{E}}_R,{\textbf{E}}_{\Theta }\}$ are standard polar coordinates and basis in referential configuration, respectively. The position vector in the spatial configuration, i.e. deformed tumor, is ${\textbf{x}}=r\,{\hat{{\textbf{e}}}}_r (\theta )$, where $\{r, \theta \}$ and $\{{\textbf{e}}_r,{\textbf{e}}_{\theta }\}$ are standard polar coordinates and basis in spatial configuration, respectively. Note that $r(R,\Theta ,t)$ and $\theta (R,\Theta ,t)$ are invertible mappings between the referential and spatial configurations. It follows that the deformation gradient ${\textbf{F}}= {\text {Grad}}{\textbf{x}}$ and the left and right Cauchy-Green deformation tensors read

21 $$\begin{aligned} {\textbf{F}}= & {} r_{,R}\,{\textbf{e}}_r \otimes {\textbf{E}}_R + r\,\theta _{,R}\,{\textbf{e}}_{\theta }\otimes {\textbf{E}}_{R} + \frac{1}{R} \left( r_{,\Theta }\,{\textbf{e}}_r + r\,\theta _{,\Theta }\,{\textbf{e}}_{\theta } \right) \otimes {\textbf{E}}_{\Theta }\,, \end{aligned}$$

22 $$\begin{aligned} {\textbf{C}}= & {} {\textbf{F}}^T{\textbf{F}}=\Biggl ( r^2_{,R} + (r\,\theta _{,R})^2 \Biggr ) {\textbf{E}}_R \otimes {\textbf{E}}_R + \left( \left(\frac{1}{R}\,r_{,\Theta }\right)^2 + \left(\frac{r}{R}\, \theta _{,\Theta }\right)^2 \right) \,{\textbf{E}}_{\Theta } \otimes {\textbf{E}}_{\Theta } \nonumber \\{} & {} + \left( \frac{1}{R}\,r_{,R}\,r_{,\Theta } + \frac{r^2}{R}\,\theta _{,R}\,\theta _{,\Theta } \right) \,({\textbf{E}}_R \otimes {\textbf{E}}_{\Theta } + {\textbf{E}}_{\Theta } \otimes {\textbf{E}}_R)\,, \end{aligned}$$

23 $$\begin{aligned} {\textbf{B}}= & {} {\textbf{F}}{\textbf{F}}^T=\Biggl ( r^2_{,R} + \frac{1}{R}\,(r_{,\Theta })^2 \Biggr ) {\textbf{e}}_r \otimes {\textbf{e}}_r + \left( \left(r\,\theta _{,R}\right)^2 + \left(\frac{r}{R}\, \theta _{,\Theta }\right)^2 \right) \,{\textbf{e}}_{\theta } \otimes {\textbf{e}}_{\theta } \nonumber \\{} & {} + \left( \frac{1}{R}\,r_{,R}\,r_{,\Theta } + \frac{r^2}{R}\,\theta _{,R}\,\theta _{,\Theta } \right) \,({\textbf{e}}_r \otimes {\textbf{e}}_{\theta } + {\textbf{e}}_{\theta } \otimes {\textbf{e}}_r)\,, \end{aligned}$$

where the subscript comma represents the partial derivative with respect to the argument. Expressing the velocity and acceleration in polar coordinates as

24 $$\begin{aligned} {\textbf{v}}= r_{,t}{\textbf{e}}_r+r \theta _{,t}{\textbf{e}}_\theta \,,\,\, {\textbf{v}}_{,t} = \left( r_{,tt} - r (\theta _{,t})^2 \right) {\textbf{e}}_r + \left( r \theta _{,t} + 2 r_{,t} \theta _{,t} \right) {\textbf{e}}_{\theta } \,, \end{aligned}$$

the Piola stress and it’s divergence has the form

25a $$\begin{aligned} \pmb {\Pi }&= \Pi _{rr}{\textbf{e}}_r\otimes {\textbf{e}}_r+\Pi _{r\theta }{\textbf{e}}_r\otimes {\textbf{e}}_\theta +\Pi _{\theta r}{\textbf{e}}_\theta \otimes {\textbf{e}}_r+\Pi _{\theta \theta }{\textbf{e}}_\theta \otimes {\textbf{e}}_\theta \,, \end{aligned}$$

25b $$\begin{aligned} {\text {Div}}{\pmb {\Pi }}&= \left( \Pi _{r r_{,r}}+\frac{1}{r} \left( \Pi _{{\theta r} _{,\theta }} + \Pi _{r r} - \Pi _{\theta \theta } \right) \right) {\textbf{e}}_r + \left( \Pi _{{r \theta }_{,r}}+\frac{1}{r} \left( \Pi _{{\theta \theta } _{,\theta }} + \Pi _{r \theta } + \Pi _{\theta r} \right) \right) {\textbf{e}}_{\theta }\,. \end{aligned}$$

The interaction between the tumor and the surrounding tissue defines the boundary condition for (12). Here, we simplify this interaction by considering uniform pressure

26 $$\begin{aligned} {\textbf{T}}\,{\textbf{n}}= -p_0{\textbf{n}}, \end{aligned}$$

where $p_0$ is the pressure due to the interaction between the tumor and the surrounding tissue, and ${\textbf{n}}$ is the normal to the boundary in spatial configuration. This simplification is valid when the surrounding tissue is much softer and its size is larger than the tumor such that their interface is not affected by the outer boundary of the tissue.

The governing equations are the balance of linear momentum and the evolution of the remodeling. The balance of linear momentum is obtained by substitution of (14), (15), (23) and (25b) into (12), and the evolution of the remodeling is obtained by substitution of (4), (19) into (10). The associated initial conditions are taken as

27 $$\begin{aligned} {\textbf{P}}^i={\textbf{I}}\,, \,{\textbf{T}}^i=-p_0 {\textbf{I}}\, , \, {\textbf{v}}^i={\varvec{0}}\, , \end{aligned}$$

and the boundary conditions are (16), where we take $p_0$ to be uniform and time independent. The system of nonlinear equations are solved numerically using the finite differences method. The referential two dimensional domain is discretized uniformly in radial and angular directions. The numerical solution algorithm starts from a system in equilibrium, such that the stress field is homogeneous with ${\textbf{T}}^i=-p_0{\textbf{I}}$, which gives rise to initial uniform elastic deformation ${\textbf{F}}^{i}_{e}$, and ${\textbf{F}}= {\textbf{F}}^{i}_{e}$. The Mandel stress (4) and the Piola stress (15) are calculated, accordingly. Next, the configuration and the remodeling fields are integrated by (12) and (10) respectively using explicit forward Euler integration. Convergence of the solution was verified by a sequence of grid refinements. This process is repeated to obtain the time integration of reconfiguration and remodeling processes. The model predictions for different nutrient fields in presented in the next section.

## Results

**Figure 2:**
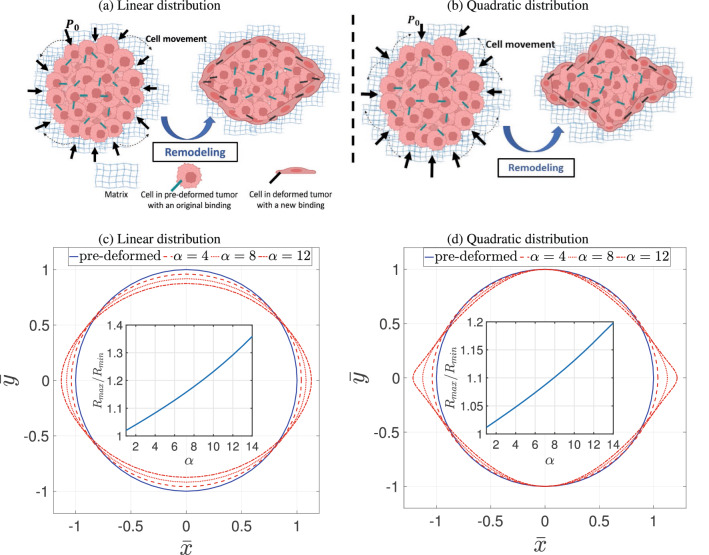
Deformation of a tumor under gradient of nutrients. A schematic representation of the remodeling in linear distribution of nutrients (**a**), leading to ellipsoidal geometry, and quadratic distribution (**b**), giving rise to irregular ellipsoidal geometry. The corresponding results of theory for $\alpha =\{4,8,12\}$, $p_0=0$ and ${\bar{t}}=1.5 \times 10^{-4}$s^2^/cm are shown in (**c**) and (**d**). The inset graphs show the ratios of vertex to covertex $R_{max}/R_{min}$ for range of $\alpha$. The rate of remodeling is shown to increase with nutrient concentration in both types distributions.

In this section, we investigate the effect of nutrient distribution on the deformation and remodeling of the tumor. For convenient we use both Cartesian and Polar coordinates to express the nutrient distribution and thus the gradient. The use of Cartesian coordinates gives better understanding of the way such distributions, or gradients, of nutrient can be practically obtained by experiment. We considered two types of nutrient distributions; linear ($c_1$) and quadratic ($c_2$) distributions in the *x* direction. This practically resembles the gradient of nutrients along the microfluidic channels of tumoroid-on-a-chip platforms.

28a $$\begin{aligned} c_1= & {} \sqrt{\alpha }\,{\bar{x}} + c_0, \qquad {\textbf{s}}_1 = \alpha \, {\varvec{i}}\otimes {\varvec{i}}\,, \end{aligned}$$

28b $$\begin{aligned} c_2= & {} \frac{\sqrt{\alpha }}{2}\,{\bar{x}}^2 + c_0, \qquad {\textbf{s}}_2 = \alpha \,{\bar{x}}\, {\varvec{i}}\otimes {\varvec{i}}\,, \end{aligned}$$

where ${\bar{x}}=x/R_0$ is normalized length measured from the center of tumor where the nutrient concentration is $c_0$, ${\varvec{i}}$ is unit vector in *x* direction, $\alpha$ is the remodeling coefficient and $R_0$ is the initial tumor radius. The corresponded representations in polar coordinates are

29a $$\begin{aligned} {\textbf{s}}_1&= \alpha \left[ \left( \cos ^2{\theta } \right) \,{\textbf{e}}_r \otimes {\textbf{e}}_r + \left( \sin ^2{\theta } \right) \,{\textbf{e}}_{\theta } \otimes {\textbf{e}}_{\theta } - \frac{\sin {2\theta }}{2} ({\textbf{e}}_r \otimes {\textbf{e}}_{\theta } + {\textbf{e}}_{\theta } \otimes {\textbf{e}}_r) \right] \,, \end{aligned}$$

29b $$\begin{aligned} {\textbf{s}}_2&= \alpha {\bar{r}}^2 \left[ \left( \cos ^4{\theta } \right) \,{\textbf{e}}_r \otimes {\textbf{e}}_r + \left( \frac{\sin ^2{2\theta }}{4} \right) \,{\textbf{e}}_{\theta } \otimes {\textbf{e}}_{\theta } - \frac{\cos ^2{\theta } \sin {2\theta }}{2} ({\textbf{e}}_r \otimes {\textbf{e}}_{\theta } + {\textbf{e}}_{\theta } \otimes {\textbf{e}}_r) \right] \,, \end{aligned}$$

where ${\bar{r}}=r/R_0$. These two cases can approximately represent two setups commonly used in tumor-on-a-chip microfluidic devices. The linear nutrient distribution (28a) represents setup with source and sink reservoirs at the two ends of the channel, while the quadratic distribution (28b) represents setup with two source reservoirs at the two ends of the channel with a constant sink in the middle. Equations (29) are substituted in (10) to find the evolution of ${\textbf{P}}$. Figure 2 shows the simulation results of the deformed boundary of tumor and the ratios of $R_{max}/R_{min}$ for different values of $\alpha$ in both linear and quadratic distributions of nutrients. Note that $R_{max}$ and $R_{min}$ are the vertex (radius of major axis) and the co-vertex (radius of minor axis) of the ellipsoidal geometry of the deformed tumor, respectively. We scale the time by the remodelling coefficient *a* to obtain the intrinsic remodeling characterization ${\bar{t}}=\rho a t$ s^2^/cm and used the following values for Lama’s constants, $\lambda =0.12$ N/cm^2^ and $\mu =0.19$ N/cm^2^ and density, $\rho _0=0.01$ kg/cm^3^^37^. As for the boundary pressure, we studied two cases. First, we set the boundary pressure $p_0=0$ N/cm^2^ to look into only residual stresses developed in the tumor due to remodeling.

**Figure 3:**
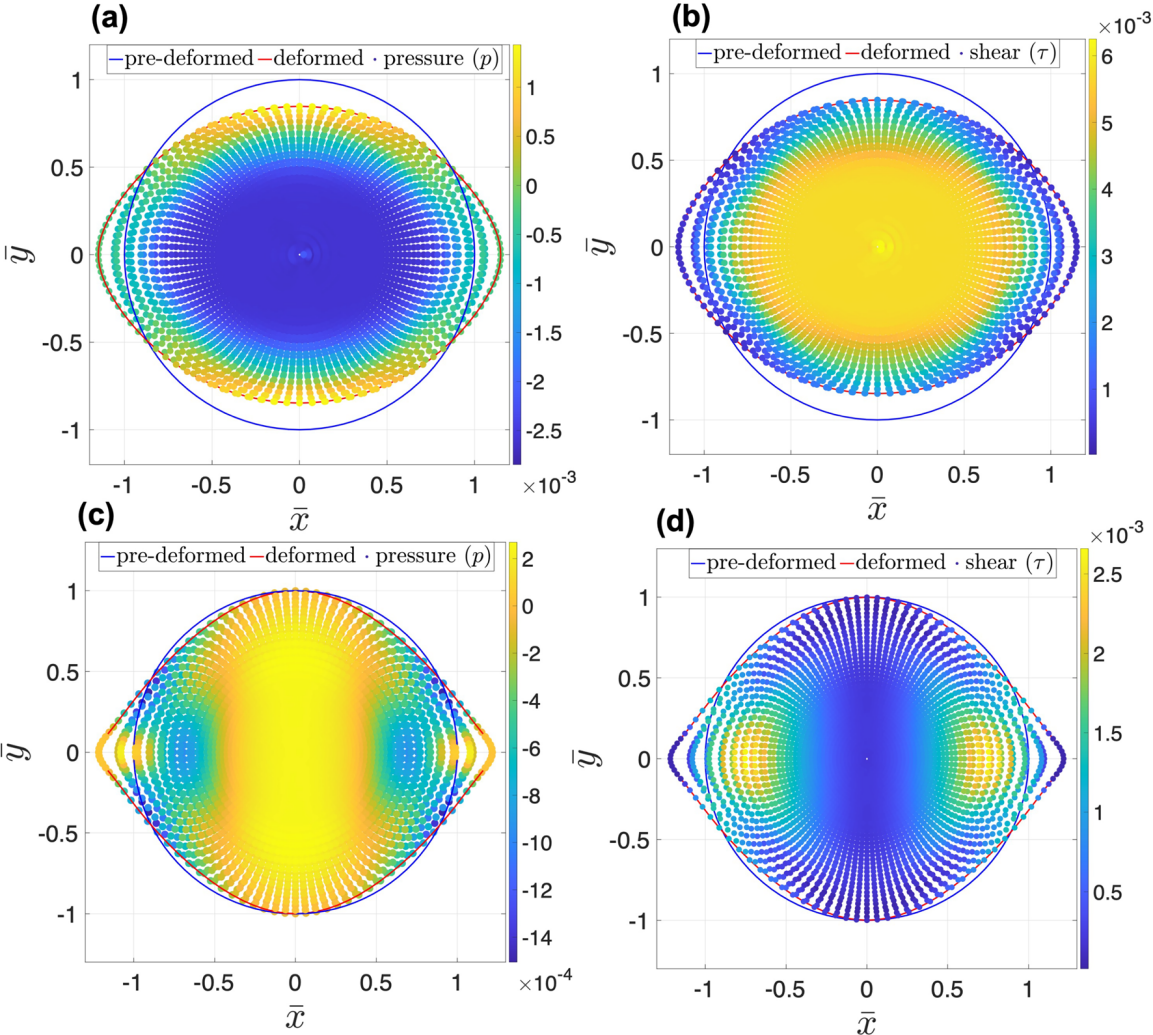
Distribution of residual pressure *p* and shear $\tau$ developed in the deformed body of tumor in response to linear and quadratic distributions of nutrients. The remodeling is obtained for $\alpha =12$, $p_0=0$ and ${\bar{t}}=1.5 \times 10^{-4}$s^2^/cm. Stress distributions are mostly homogeneous in linear nutrient distribution, indicating homogeneous remodeling. In quadratic distribution of nutrient, the stress distribution is inhomogeneous, vanishing around the covertex region. However, stress concentration is observed at the vertex edges indicating significant local remodeling.

The remodeling occurs when cancer cells at the outer layer of tumor acquire elongated morphology and change their local cell-cell and cell-matrix bindings, Fig. 2a,b. This heterogeneity in cells behaviour exhibiting circumferentially aligned cells was shown to be correlated with the mechanical stress field^38^. As can be seen in Fig. 2c,d, the presents of nutrient enhance remolding, that is, the rate of remodeling increases with nutrient concentration for both linear and quadratic distributions. However, the shapes of the tumours are different for the two cases. In linear distribution, the spherical tumour remodels into an ellipsoidal shape, where extension and perpendicular contraction occur along the vertex and covertex, respectively. In quadratic distribution, spherical tumour remodels into a irregular ellipsoidal shape with sharper edges along the vertex and no deformation along the covertex. These differences are contributed to the different remodeling fields which are proportional to nutrient gradient, hence, for the linear case, the remodeling is homogeneous, while for the quadratic distribution, the remodeling is inhomogeneous and increasing with $|{\bar{x}}|$. The ratio of $R_{max}/R_{min}$ is also depicted in this figure for a range of $\alpha$ where the linear distribution imposes larger ratios compared to the quadratic. This is also supported by the analysis of the residual stress developed in the tumor due to remodeling. Figure 3 depicts the pressure *p* and the magnitude of shear stress $\tau$ evaluated by

30a $$\begin{aligned} p= & {} -\frac{1}{2} \left( {\textbf{T}}\cdot {\textbf{I}}\right) \end{aligned}$$

30b $$\begin{aligned} \tau= & {} \sqrt{ \pmb {\tau } \cdot \pmb {\tau } } \; , \end{aligned}$$

where $\pmb {\tau } = {\textbf{T}}+ p {\textbf{I}}$ is deviatoric part of the stress tensor. The pressure and the shear stress are invariant measures of the stress and are known to be mechanical stimuli that induce biological responses such as autophagy^39^ . It has been observed that direct compression of cancer cells can affect gene expression, hence alter the tumor invasiveness, rate of cancer cell proliferation and death. Shear stress, on the other hand, has less effect on the growth rate, but alters autophagy and apoptosis. Readers are referred to^39^ for details of the role of different types of stresses on cell behaviour and the associated signaling mechanochemical pathways. Figure 3 shows that for the linear distribution of nutrients the stress is homogeneous indicating homogeneous remodeling due to constant gradient of nutrients. However, for the quadratic case, the stress is inhomogeneous where limited remodeling occurs about the co-vertex, ${\bar{x}}=0$, and yields vanishing stresses around that region. This is due to zero gradient of nutrients across the co-vertex. On the other hand, stress concentration is observed at the vertex edges indicating significant local remodeling due to the local increase in the gradient of nutrients. Moreover, the vertex edges are sharper due to the high gradient of nutrients.

**Figure 4:**
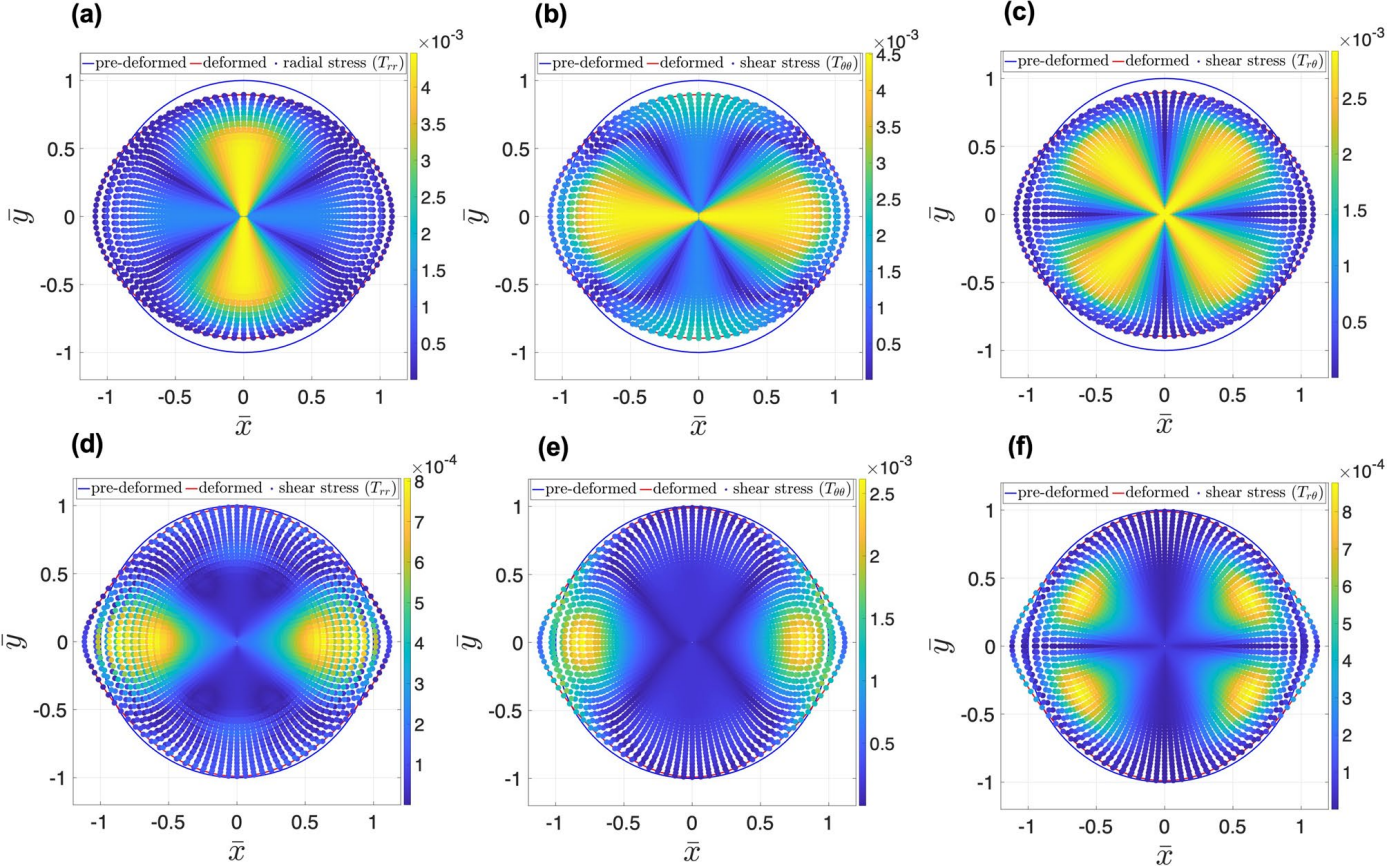
Distribution of radial $T_{rr}$, tangential $T_{\theta \theta }$ and shear $T_{r \theta }$ stresses in deformed body of tumor in response to linear and quadratic distribution of nutrients, for $\alpha =12$, $p_0=0.001$ and ${\bar{t}}=1.5 \times 10^{-4}$s^2^/cm. Radial and tangential stresses are maximum along the covertex and vertex, respectively, in linear nutrient distribution. However, the shear stress is maximum along the diagonals. In quadratic nutrient distribution, both radial and tangential stresses are maximum around the edges of the covertex where the nutrient gradient is maximum, and zero along the vertex where nutrient gradient is zero. Similar to the linear distribution, maximum shear stress in quadratic distribution is seen along the diagonals but only at the edges.

Next, we looked into non-zero pressure boundary condition. The interaction between tumor and the surrounding tissue is an important source of mechanical stresses that can trigger biological mechanisms. Different types of tumors, and their surrounding tissues, have different mechanical properties. For instance, human colon adenocarcinoma, glioblastoma and osteosarcoma are types of solid tumors with different mechanical stiffnesses^40^. Besides, acute myeloid leukemia (AML) is generally considered a liquid tumor. The interaction at the interface of tumor and tissue gives rise to stresses inside the tumor. To model a realistic boundary condition, one may model the tissue as a hyperplastic material similar to the tumor, and consider appropriate interaction between the tumor and the tissue, such as zero jumps in displacement and traction across the interface. Here, we simplify this interaction by considering a uniform pressure on the boundary of a tumor. Figure 4 shows the radial, tangential and shear stresses developed in the deformed body of tumor in response to linear and quadratic distributions of nutrients and in presence of constant boundary pressure. In linear nutrient distribution, the radial and tangential stresses are maximum along the covertex and vertex, respectively. The shear stress is maximum along the diagonals. However, in quadratic nutrient distribution, both radial and tangential stresses are maximum around the edges of the covertex where the nutrient gradient is maximum, and zero along the vertex where nutrient gradient is zero. Also, the shear stress is maximum at the edges of the diagonals. As can be seen, the nutrient distribution strongly changes the pattern of stress distributions.

**Figure 5:**
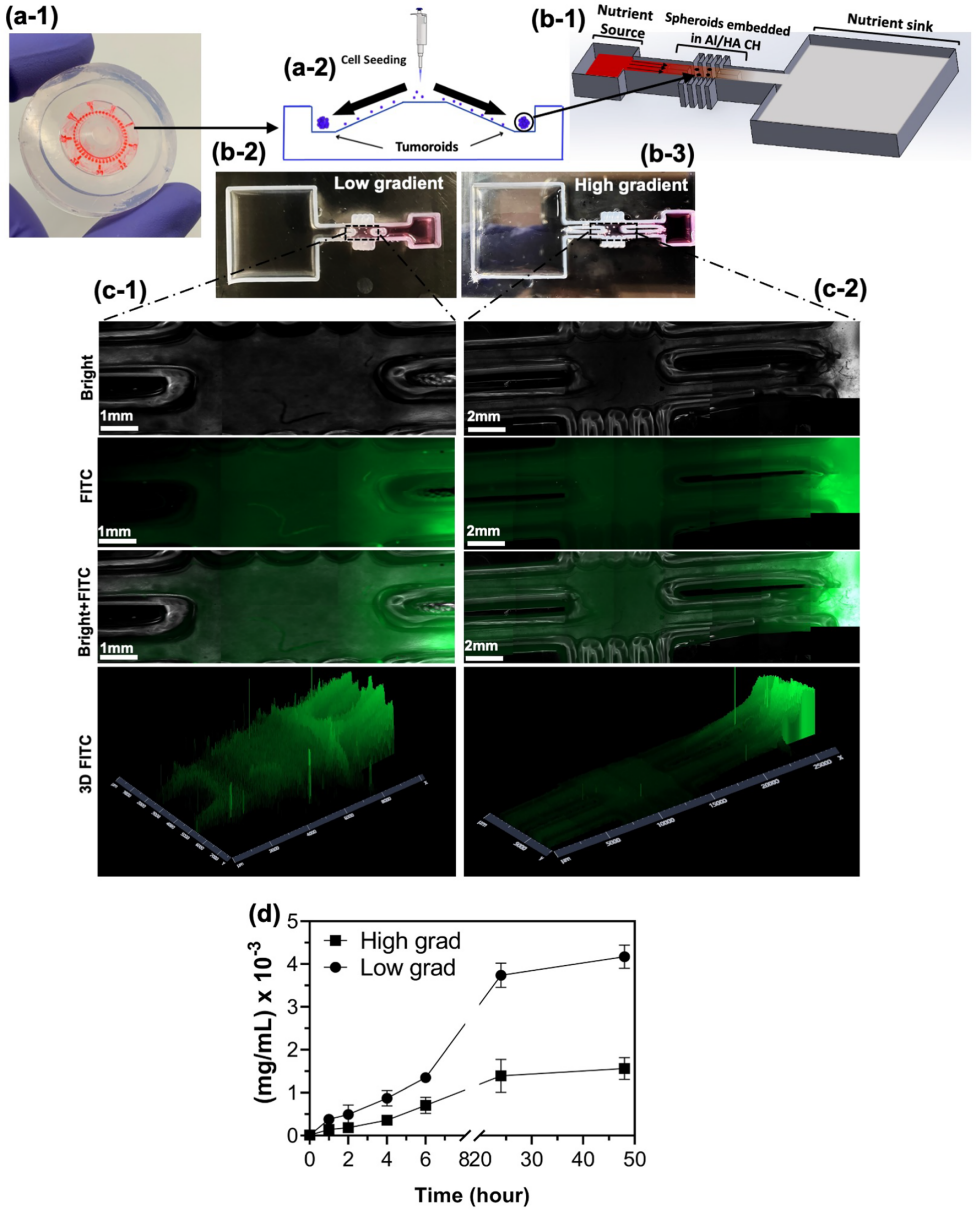
EZ-seed culture plate (**a-1**) and the schematic of the microwells (**a-2**) are shown. GB tumoroids were generated by seeding single cell suspension in the microwell array. The schematic of open-surface tumoroid-on-a-chip model that induces gradient of biochemicals is shown in (**b-1**). The chip consists of a central chamber for the GB tumoroid-embedded CH connected to a sink and a source reservoirs. To generate high and low gradients, short (**b-2**) and long (**b-3**) channels were designed to vary the diffusion length. Diffusion of FITC through the CH in channels was imaged, (**c-1**) and (**c-2**), and measured over time, (**d**), indicating lower diffusion (higher gradient) in long channels and higher diffusion (lower gradient) in short channels.

To evaluate the presented theory and simulation results, we designed an experimental model to resemble tumor remodeling. Glioblastoma (GB) multiforme was selected to investigate the remodeling capability. GB multiforme accounts for 47.1% of malignant tumors in the central nervous system^41^. Highly malignant GB tumors grow and spread rapidly in the CNS and hence significantly affects the patient’s physical and cognitive abilities^42^. The importance of better understanding their biomechanical responses makes them a proper candidate for remodeling analysis. To investigate the remodelling behaviour of the GB tumoroids in response to biochemical stimuli, an in-vitro GB tumoroid-on-a-chip model was designed and fabricated. GB tumoroids were generated using EZ-seed culture plate, Fig. 5a-1,a-2. We used an extrusion 3D bioprinter and printable PDMS resin to fabricate a microfluidic chip with three compartments including a central tumor chamber, where the GB tumoroid-embedded CH was injected, and the two side chambers as the nutrient source and sink reservoirs, as shown in Fig. 5b-1. The chip was capable of generating gradient of biochemicals and incorporates GB tumoroids embedded inside the hyaluronic acid (HA)/alginate composite hydrogel (CH)^43^. We replaced matrigel with HA to avoid the common finger-type invasion of the GB tumoroids, while providing freedom for tumor remodeling within the ECM. The difference between the volume of chambers guarantees the gradient of biochemical through the central chamber. As the platform had no control on producing different types of gradient, we focused on generating different levels of gradient for which we fabricated short and long diffusion channels inside the tumor chamber, shown in Fig. 5b-2,b-3, respectively. Chamber with longer channels can contain more amount and CH, leading to longer diffusion length and hence higher gradient of nutrient. To evaluate the diffusion rate and the generated gradient, the transport profile of fluorescein isothiocyanate-Dextran, (FITC-Dextran, 20 kDa), was measured in both chips. FITC-Dextran solution (1 mg/mL in PBS) was added to the source chambers while the sink chambers were filled with only PBS Fig. 5c-1,c-2. To quantify the overall diffusion, 20 μL of the sink chambers were sampled every two hours and the fluorescent intensity of the diffused FITC was measured using Nano plate reader. The change in the concentration of FITC over 48h is shown in Fig. 5d. As can be seen, higher concentration of FITC was diffused in low gradient chip, while the high gradient chip allowed less diffusion but sustained higher level of gradient.

**Figure 6:**
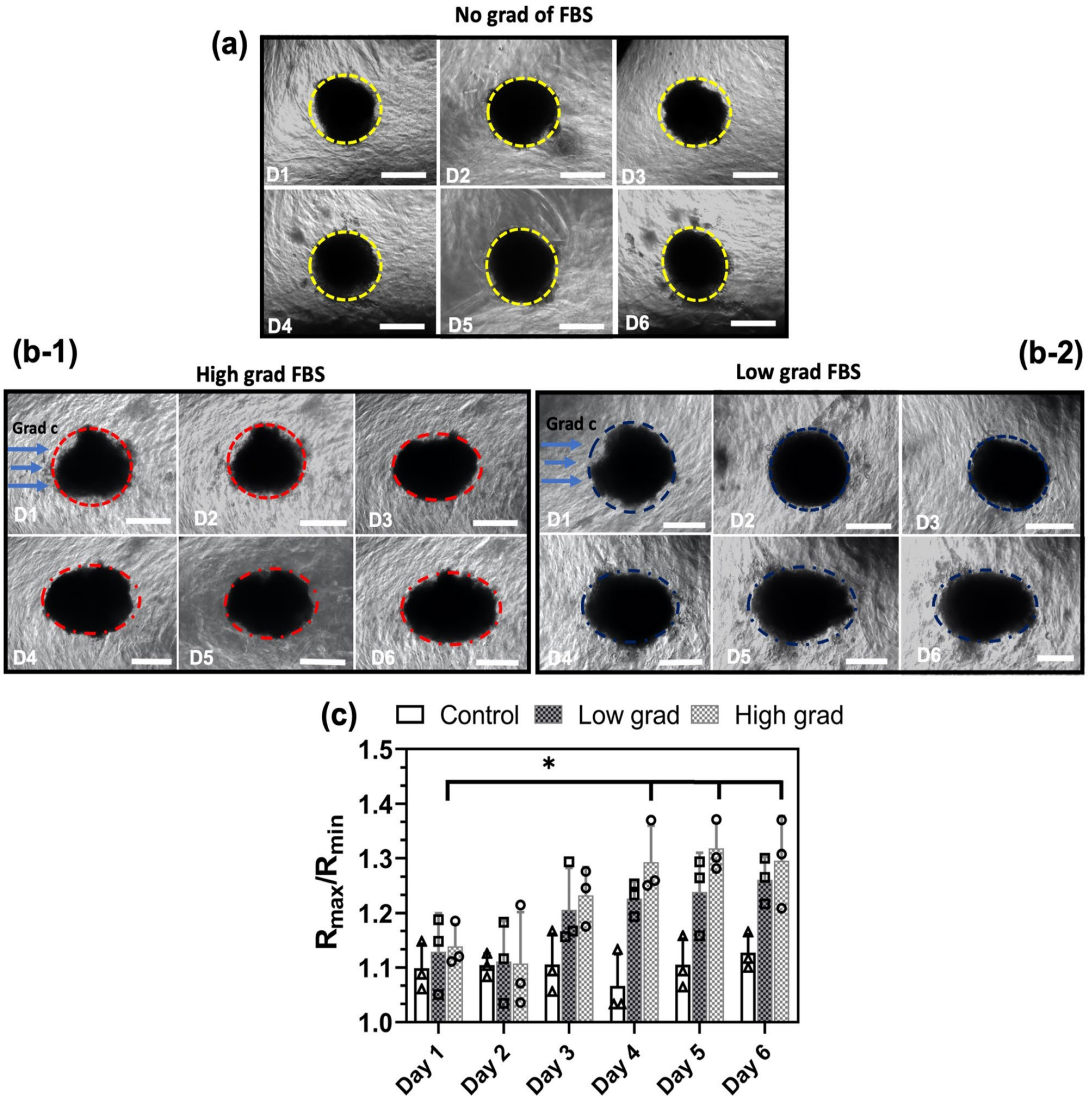
Asymmetric remodeling of GB tumoroids in response to high gradient (**b-1**) and low gradient (**b-2**) of FBS was imaged over 6 days and compared with the control (**a**). Spherical tumoroids remodeled to ellipsoidal shapes along the direction of FBS gradient. Measurement of vertex to covertex ratios R$_{max}$/R$_{min}$ of tumoroids indicated that higher gradient gives rise to higher levels of remodeling (**c**). Scale bares are 200 μm.

Figure 6 depicts the remodeling of tumoroids in response to the zero (a), high (b-1) and low (b-2) gradients of FBS. A chip with source and sink chambers of the same size, both filled with the same concentration of FBS and saturated CH with the diffused FBS was used as a control. Tumoroids were imaged over 6 days and the vortex to co-vortex ratios (R$_{max}$/R$_{min}$) were measured (c). As can be seen, tumoroids were able to respond to the gradient of FBS and undergo remodeling configuration. Higher gradient of FBS (high gradient *vs*. low gradient chip) gave rise to higher ratios (R$_{max}$/R$_{min}$) indicating that gradient of nutrient is indeed the characteristic property of the biochemical stimuli.

To investigate the effect of remodeling on cell distribution within tumoroids, IHC technique was used to stain the interior part of tumoroids.

**Figure 7:**
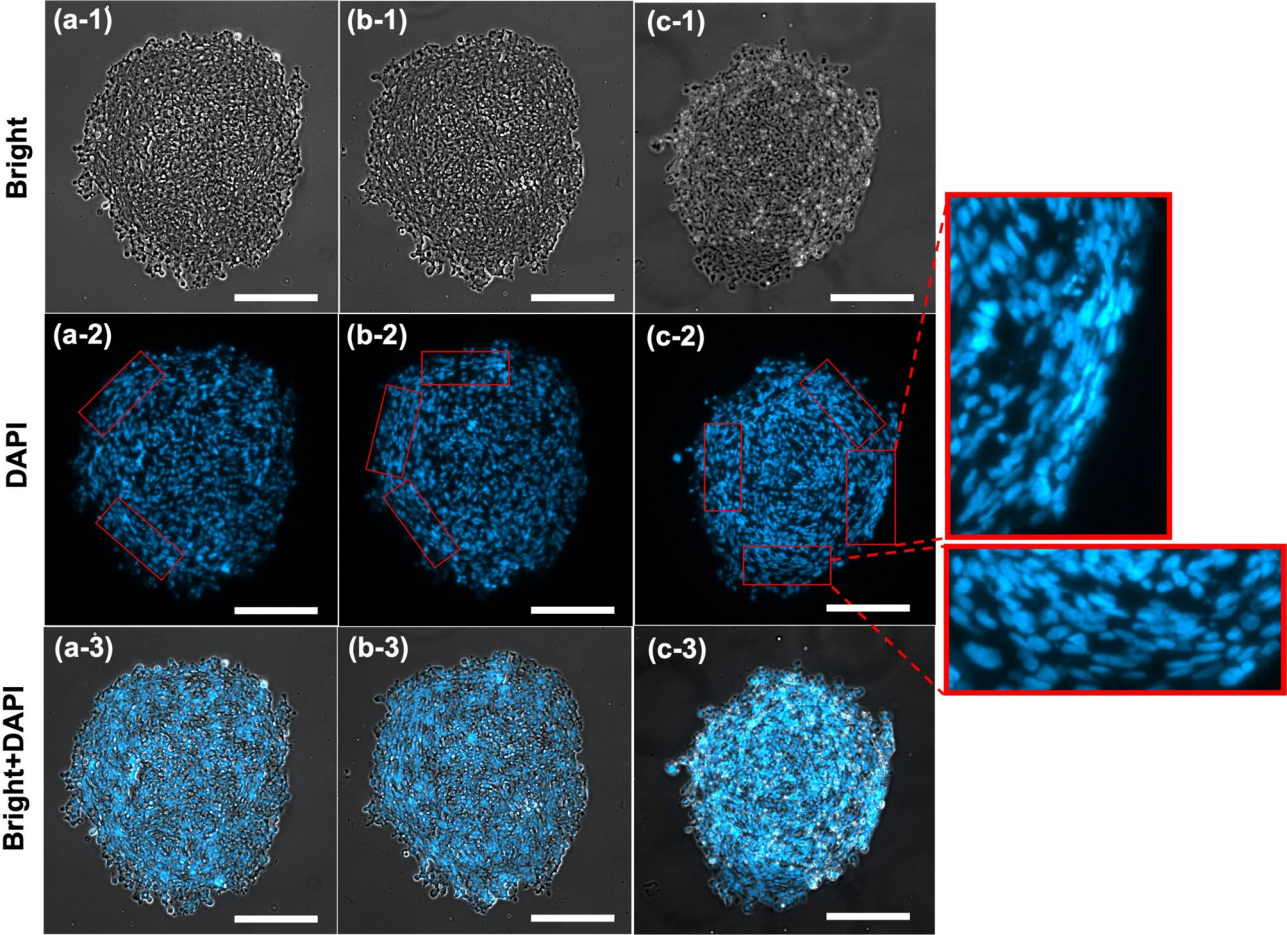
Remodeling gives rise to heterogeneity in cell distribution within tumoroids. Cells alignment in cross sections of GB tumoroids with low (**a**) to high (**c**) degrees of ellipticity. More alignment zones were observed in (**c**) indicating the correlation between the degree of remodeling and level of cell alignment. Higher alignments were identified at the circumferential regions of tumoroids where the cells undergo larger displacement and hence tend to further align with themselves. Cells in interior regions have no alignment preference, therefore, acquire isotropic orientation. Scale bars are 200 μm.

Figure 7 shows the cross-sections of three tumoroids with different degrees of ellipticity due to remodeling. The tumoroid with higher degree of ellipticity (c) contained more circumferentially aligned cells compared to less ellipsoidal tumoroid in (a) and (b). These cells undergo larger displacement compared to the interior cells, predicted by model and shown in Figure 2(c) and (d), as the tumoroid remodels to an ellipsoidal shape. This is depicted in the magnified zones marked by red boxes which qualitatively show the correlation between the degree of remodeling and level of cell alignment. Due to the movement schematically illustrated in Fig. 2a,b, these cells tend to align with themselves as the remodeling takes place. This pattern identifies a heterogeneity in cell distribution that is corresponded to the remodeling in response to the gradient of biochemical stimuli.

## Discussion

In this work, we studied asymmetric remodeling of tumor in response to biomechanical stimuli. We considered the tumor body as a simple evolving material, i.e. defined by their strain energy function, with a volume-preserving isotropic remodeling evolution. A characteristic tensor associated to the biomechanical stimuli, e.g. gradient of nutrient, was proposed that derives the remodeling. In this framework, principal balance laws were considered for tumor mass and linear momentum. As for the boundary conditions, we studied zero pressure, to investigate the residual stresses due to only the remodeling, and constant pressure to model the tumor-tissue interaction. Two types of nutrient distributions, linear and quadratic, were considered to explore the remodeling response. Results showed that the nutrient distribution strongly changes the remodeling and the stress distributions. Using the experimental platform, we were able to validate our hypothesis that the gradient of nutrient is the main characteristic of nutrient distribution in deriving the remodeling. In addition, we showed that the degree of remodeling is proportional to the magnitude of gradient. It was observed that the remodeled shape and the stress distribution are respectively ellipsoid and homogeneous, while irregular ellipsoid and in-homogeneous, in linear ad quadratic distributions of nutrient, respectively. To evaluate the model, an in-vitro open surface GB tumoroid-on-a-chip platform was designed and fabricated. Tumoroids were embedded in a HA/alginate composite hydrogel, injected inside the chip and exposed to gradient of FBS. Results showed that tumoroids were influenced by the gradient of FBS and undergo asymmetric remodeling. Also, measurement of vertex to covertex ratios over time indicated that higher gradient of FBS induces higher degree of ellipticity. It was also observed that remodeling induced heterogeneity in cell alignment forming circumferentially aligned cells within the tumors. The presented framework gives a new insight to better understanding the interrelationship between the mechanical stress and biological responses in tumors. Using the proposed model, we were able to study the role of gradient of nutrient, as one of the key factors influencing tumor progressions, on biomechanics of tumor including tumor deformation, asymmetric remodeling, and stress distribution. Future research directions include, but not limited to, experimental measurements of the correlation between the distribution of stresses and the biological responses, such as cell proliferation, autophagy and apoptosis, and calibration of model parameters, such *a* and $p_0$, using quantitative biological assays. Another promising research direction can be utilizing the proposed framework to study a more realistic model that includes heterogeneity in terms of cell population and phenotype, and ECM effects.

## Methods

### Cell and tumoroid culture

Human glioblastoma cancer cells (U251) were cultured in Dulbecco’s Modified Eagle Medium (DMEM) supplemented with 10% (v/v) fetal bovine serum (FBS), and 1% (v/v) Penicillin/Streptomycin. The U251 cells were incubated at 37$^\circ$C in a humidified atmosphere of 5% CO2, and at 90% confluency were trypsinized with Gibco^TM^ Trypsin-EDTA (0.5%) into a single cell suspension. The cell suspension was centrifuged at 300g (5 minutes). After removing the supernatant, cells were suspended in 1 mL of medium and counted using a standard hemocytometer. EZ-seed culture plate from Apricell biotechnology (https://www.apricell.com/) was used to generate U251 tumoroids. 500 μL of 1 × 10^6^ cells/mL cell suspension was loaded dropwise through the guiding channels and was kept in an incubator for 10 minutes to let the cells fill the microwells. Afterwards, the culture medium was aspirated and exchanged with a new medium. U251 tumoroids were monitored over 4 days until the formation stage is completed and they start the volumetric growth with average diameter of 500 μm.

### Tumoroid-on-a-chip model

To prepare the GB tumoroids embedded in CH, tumoroids were removed from SFMAs by gentle aspiration of the media from the loading zone of the microwell array and collected in a Petri dish. To make 500 μL of CH, 62.5 μL of 5mg/mL alginate, 200 μL of 4mg/mL HA and 234.5 μL of DMEM were mixed and crosslinked with 3 μL of 1M CaCl_2_. The CH solution was pipetted inside the central chamber of the microfluidic chips. GB Tumoroids in 2 uL media were pipetted inside the CH at the center of the tumor chambers. The source and sink reservoirs were filled with basal media. 10% FBS was added to the source chambers while the sink chambers were kept FBS-free. Every 24h the source chambers were refilled with fresh media containing 10% FBS and the sink chambers refilled with FBS-free media.

### IHC staining

Tumoroids were fixed with 10% neutral buffered formalin for 45 minutes, washed twice with Dulbecco’s phosphate-buffered saline (DPBS), embedded in 2% agarose solution and kept in room temperature for 30 min. Agarose-embedded tumoroids were dehydrated by 10 min of submerging in 70%, 90% and 100% ethanol, respectively, followed by twice submerging in 100% Xylene for 20 min. Samples were then embedded in melted paraffin wax for four hours and placed in cassette for sectioning. Paraffin blocks were then sliced to approximately 5 μm thickness using microtome device and deparaffinized by two minutes wash with 100% Xylene, 100% ethanol, 90% ethanol and distilled water, respectively. Tumoroids sections were then counterstained with 4’, 6-diamidino-2-phenylindole (DAPI) and imaged using fluorescent microscopy.

### Statistical analysis

All experiments were repeated three times and the average and the standard deviation are reported. Significance analysis was performed using two-way ANOVA analysis. Differences were considered statistically significant at P-value < 0.05.

## Data Availability

The datasets generated for the current study are available upon the publication.
